# Post-anthesis dry matter and nitrogen accumulation, partitioning, and translocation in maize under different nitrate–ammonium ratios in Northwestern China

**DOI:** 10.3389/fpls.2024.1257882

**Published:** 2024-03-19

**Authors:** Bing Wu, Zhengjun Cui, Effah Zechariah, Lizhuo Guo, Yuhong Gao, Bin Yan, Hongsheng Liu, Yifan Wang, Haidi Wang, Li Li

**Affiliations:** ^1^ College of Life Science and Technology, Gansu Agricultural University, Lanzhou, China; ^2^ State Key Laboratory of Aridland Crop Science, Lanzhou, China; ^3^ College of Agronomy, Tarim University, Alar, China; ^4^ Council of Scientific and Industrial Research (CSIR)-Plant Genetic Resources Research Institute, Bunso, Ghana; ^5^ College of Agronomy, Gansu Agricultural University, Lanzhou, China; ^6^ Huining Promotion Center of Agricultural Technology, Huinning, China; ^7^ Baiyin Promotion Center of Agricultural Technology, Baiyin, China

**Keywords:** mixed NO_3_^−^/NH_4_^+^, maize, grain yield, enzymatic activity, dry matter and nitrogen partitioning and transportation

## Abstract

**Introduction:**

An appropriate supply of ammonium (NH4+) in addition to nitrate (NO3−) can greatly improve plant growth and promote maize productivity. However, knowledge gaps exist regarding the mechanisms by which different nitrogen (N) fertilizer sources affect the enzymatic activity of nitrogen metabolism and non-structural carbohydrates during the post-anthesis period.

**Methods:**

A field experiment across 3-year was carried out to explore the effects of four nitrateammonium ratio (NO3−/NH4+ = 1:0 (N1), 1:1 (N2), 1:3 (N3), and 3:1 (N4)) on postanthesis dry matter (DM) and N accumulation, partitioning, transportation, and grain yield in maize.

**Results:**

NO3-/NH4+ ratio with 3:1 improved the enzymatic activity of N metabolism and non-structural carbohydrate accumulation, which strongly promoted the transfer of DM and N in vegetative organs to reproductive organs and improved the pre-anthesis DM and nitrogen translocation efficiency. The enzymatic activities of nitrate reductase, nitrite reductase, glutamine synthetase, glutamine oxoglutarate aminotransferase, and non-structural carbohydrate accumulation under N4 treatment were increased by 9.30%–32.82%, 13.19%–37.94%, 4.11%–16.00%, 11.19%–30.82%, and 14.89%–31.71% compared with the other treatments. Mixed NO3−-N and NH4+-N increased the total DM accumulation at the anthesis and maturity stages, simultaneously decreasing the DM partitioning of stem, increasing total DM, DM translocation efficiency (DMtE), and contribution of pre-anthesis assimilates to the grain (CAPG) in 2015 and 2017, promoting the transfer of DM from stem to grain. Furthermore, the grain yield increased by 3.31%–9.94% (2015), 68.6%–26.30% (2016), and 8.292%–36.08% (2017) under the N4 treatment compared to the N1, N2, and N3 treatments.

**Conclusion:**

The study showed that a NO3−/NH4+ ratio of 3:1 is recommended for high-yield and sustainable maize management strategies in Northwestern China.

## Introduction

Nitrogen is one of the most important nutrients that affect plant growth, development, and yield (Sun et al., 2014). It was found that two main forms of N were used by plants under natural conditions: nitrite N (NO_3_^−^-N) and ammonia N (NH_4_^+^-N), although some plants can absorb it in the form of urea or amino acids, while both NH_4_^+^-N and NO_3_^−^-N are effective and can be directly absorbed by plants (Isaiah et al., 2021; Moussa et al., 2021). In addition to the application amount, the form of usable N has a significant impact on the growth, photosynthesis, yield, and quality of crops (Ali et al., 2013). It follows that matching plant preference for N form and soil nitrogen dynamics can improve nitrogen use efficiency and yield (Zhang et al., 2016). Most plants responded better to mixed NO_3_^−^-N and NH_4_^+^-N than solitary NO_3_^−^-N or NH_4_^+^-N (Zhou et al., 2011). The preferred N form (NH_4_^+^ and NO_3_^−^) for crop growth varies depending on the genotype of an individual crop species, environmental conditions, developmental stage, and total N concentration supplied (Guo et al., 2012; Sun et al., 2014). The predilection of crops for N forms may be influenced by the NO_3_^−^/NH_4_^+^ ratio in the habitat from which they originated (Andrews et al., 2013).

Maize (*Zea mays* L.), one of the most crucial crop species in the world (Qian et al., 2018), is widely planted worldwide for food, fodder, and industrial use, both in tropical and temperate regions (Calderón-Vázquez et al., 2011). Maize develops on well-ventilated dryland soil, where NO_3_^-^ is generally the dominant and is also the dominant N uptake form owing to soil nitrification (Wang et al., 2018). Maize has also been reported to have a higher preference for ammonia N than nitrite N (George et al., 2016; Zhang et al., 2019). NH_4_^+^-N may restrict maize seedling growth and NO_3_^−^ supply can increase the dry matter accumulation of maize at the seedling stage (Xue et al., 2016). According to a study by Sabir et al. (2013), N form had a substantial impact on shoot dry weight, leaf stomatal conductance, transpiration, and photosynthetic rate in maize shoots.

Although there were a lots of research progress achieved to understanding the mechanisms of maize absorption and assimilation NO_3_^−^-N and NH_4_^+^-N (Ma et al., 2015; George et al., 2016; Zhang et al., 2019). Furthermore, the exact mechanism underlying nitrogen metabolism is unclear at this stage, and the optimal NO_3_^−^-N to NH_4_^+^-N ratio remains to be identified. Specifically, the aims of this study were as follows: (1) to clarify the effects of nitrate–ammonium ratio on post-anthesis dry matter and nitrogen accumulation, partitioning, and translocation efficiency, (2) to explore the characteristics of the enzymatic activity and total non-structural carbohydrates, and (3) to reveal the mechanism of maize grain yield formation under different nitrate–ammonium nitrogen ratio treatments. This study will help understand the response of maize yield formation to the nitrate–ammonium ratio, and provide scientific basis for promoting sustainable development of maize in dry farming areas of the Loess Plateau.

## Materials and methods

### Study site description

**Figure 1:**
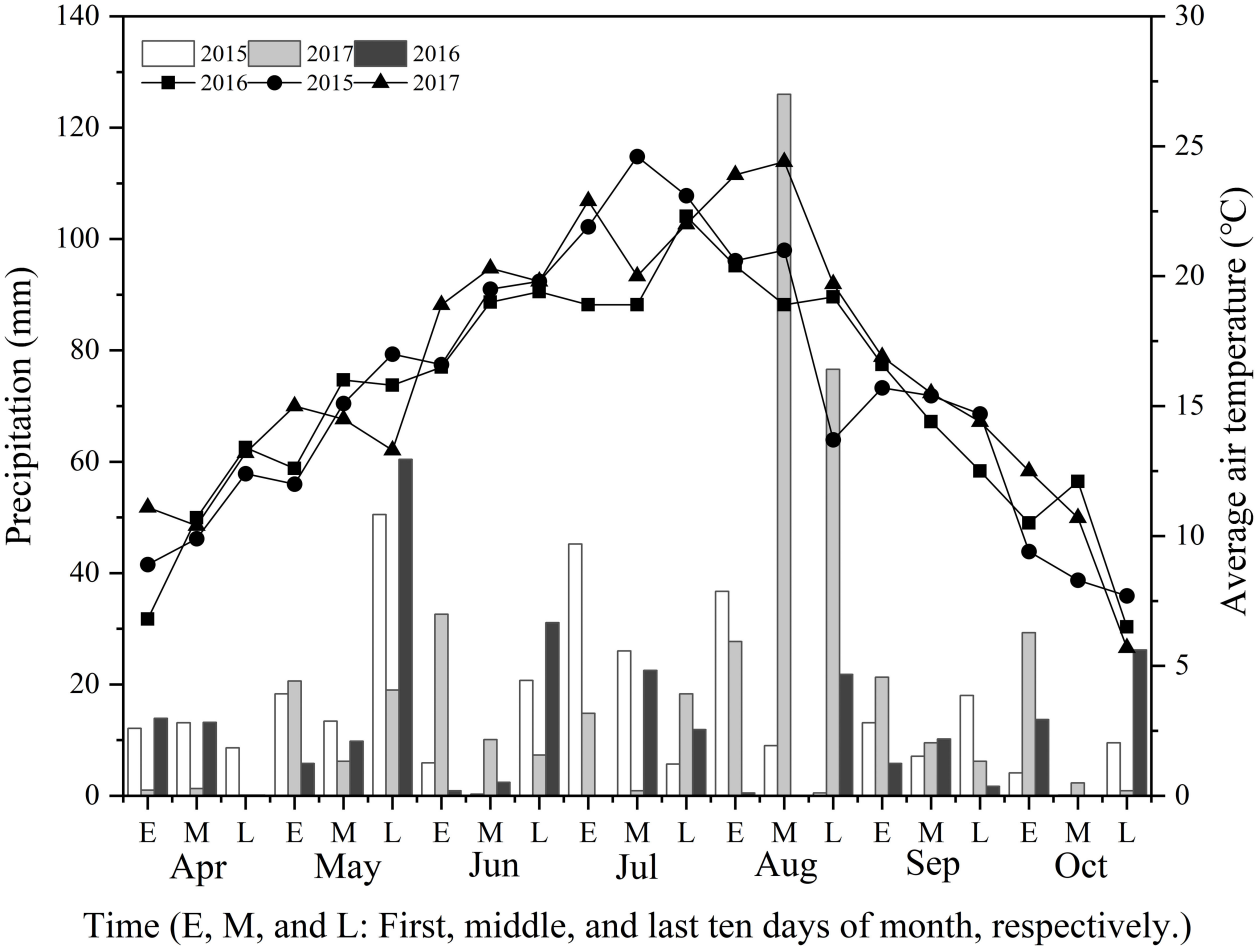
The precipitation and air temperature at the experimental site from April to October in 2015, 2016, and 2017.

The trials were conducted in 2015, 2016, and 2017 in Huishi Town, Huining County, Gansu Province, China (105°02’E, 35°38’N). The experimental location was at an altitude of 1,772 m. The study site had an average annual temperature of 8.3°C and mean annual rainfall of 356.70 mm. The topsoil (0 cm–30 cm depth) before sowing in 2015 had the following properties: pH, 8.12, 11.98 g organic matter kg^−1^, 0.97 g total N kg^−1^, 60.01 mg available P kg^−1^, and 5.39 mg available K kg^−1^. **Figure 1** depicts the meteorological data of the Huining Observatory for the three years. The rainfall in the three growing seasons was 318 mm, 252 mm, and 432 mm, respectively. The mean maximum air temperature ranges were 12.7°C–30.3°C in 2015, 15.3°C–33.3°C in 2016, and 17.0°C–34.6°C in 2017. Rainfall in April and May 2015 and 2016 was higher than that in April and May 2017, and considerably lower in August 2015 and 2016 than in 2017 (**Figure 1**). Significantly higher temperatures were reported in July 2016 and 2017 than in 2015, and higher temperatures were noted in 2016 than in 2015 and 2017 (**Figure 1**).

The 3-year trials were carried out with three replicates, where the treatments were four nitrate–ammonium ratios (**Table 1**): NO_3_^−^/NH_4_^+^ with 1:0 (sole NO_3_^−^-N, N1), 1:1 (N2), 1:3 (N3), and 3:1 (N4). Except for the form of nitrogen, the total nitrogen application rate for all treatments was consistent, all of which were 300 kg N ha^−1^ in 2016 and 2017 (180 kg N ha^−1^ in 2015; Li et al., 2017a; details of the treatments and fertilizer application amount at different periods in **Tables S1**, **S2**). The N fertilizer was applied to the soil three times: the first application was incorporated with a tandem harrow disc to a depth of 10 cm as basal N before sowing (180 kg), the second was applied at the jointing stage (60 kg), and the third was applied at the filling stage (60 kg); N Fertilizer was applied 10 cm from the maize plant to a depth of about 10 cm using a hand-held topdressing tool. 3,4-Dimethylpyrazole phosphate (DMPP) is a novel nitrification inhibitor with excellent performance, and the dosage of 0.5 kg ha^−1^–1.5 kg ha^−1^ DMPP are sufficient to achieve the best nitrification inhibition (Zerulla et al., 2001). The nitrification inhibitor DMPP (Hubei Shuangyan Chemical Co., Wuhan, China) was applied to the N2, N3, and N4 treatments to avoid the conversion of NH_4_^+^ to NO_3_^−^ during the experiment, and the dosage, inhibitor time, and amount of DMPP were explained in detail in our previous study (Cui et al., 2022). Additionally, 180 kg of 16% CaP_2_O_5_ ha^-1^ fertilizer was applied (Li et al., 2017a). In all treatments, the total K fertilizer remained constant with the addition of potassium sulfate (K_2_SO_4_), if necessary (**Tables 1**, **2**).

**Table 1:** Details of the treatments.

Treatment	Symbol	Fertilizer amount			DMPP (kg ha^−1^)^‡^
		KNO_3_ (kg ha^−1^)	(NH_4_)_2_SO_4_ (kg ha^−1^)	K_2_SO_4_ (kg ha^−1^)	
KNO_3_ fertilizer	N1	2,221.50	0.0	0.0	0.0
KNO_3_:(NH_4_)_2_SO_4_ with ratio^†^ 1:1	N2	1,110.75	707.10	956.55	1.5
KNO_3_:(NH_4_)_2_SO_4_ with ratio^†^ 1:3	N3	555.45	1,060.65	1,434.75	2.3
KNO_3_:(NH_4_)_2_SO_4_with ratio^†^ 3:1	N4	1,666.05	353.55	478.50	0.8

^†^ratios are given as NO_3_^-^/NH_4_^+^ and refer to pure N content, ‡ The application of DMPP was calculated as 1% of pure N in NH_4_^+^-N content of the basic fertilizer.

**Table 2:** Fertilizer application amount at different periods.

Treatment	KNO_3_ (N 13.5%) (kg ha^−1^)			(NH_4_)_2_SO_4_ (N 21.21%) (kg ha^−1^)			K_2_SO_4_ (kg ha^−1^)			DMPP (kg ha^−1^)		
	Before sowing	Jointing	Filling	Before sowing	Jointing	Filling	Before sowing	Jointing	Filling	Before sowing	Jointing	Filling
N1	1,332.9	444.3	444.3	0	0	0	0	0	0	0	0	0
N2	666.45	222.15	222.15	424.26	141.42	141.42	573.93	191.31	191.31	0.9	0.3	0.3
N3	333.27	111.09	111.09	636.39	212.13	212.13	860.85	286.95	286.95	1.38	0.46	0.46
N4	999.63	333.21	333.21	212.13	70.71	70.71	287.1	95.7	95.7	0.48	0.16	0.16

DMPP, 3,4-Dimethylpyrazole phosphate.

**Figure 2:**
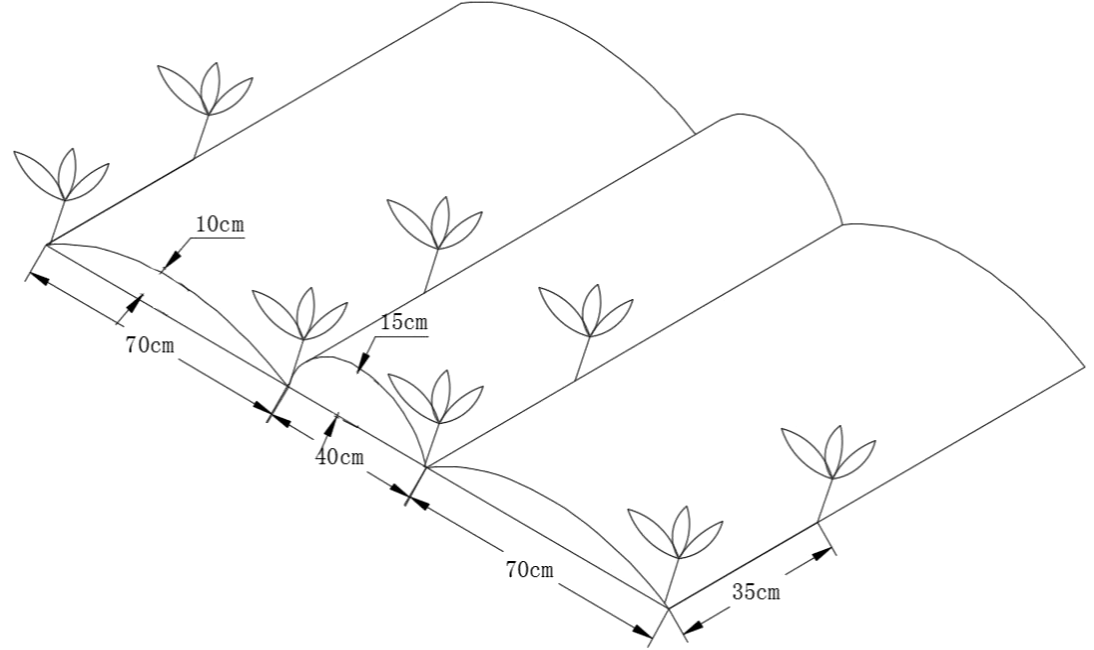
Schematic diagram of the field layout where maize plants were separated by alternating distance.

Each block had an area of 40.26 m^2^ (6.1 m × 6.6 m), consisting of narrow ridges (15 cm high and 40 cm wide) alternated with wide ridges (10 cm high and 70 cm wide) (**Figure 2**). All ridges were covered with transparent plastic polyethylene film of 0.01 mm thickness that was 140 cm wide (Lanzhou Green Garden Corp., Lanzhou, China). A ridge-furrow plastic film mulching pattern was used to reduce evaporation losses and increase the amount of rainwater collected (Liu et al., 2009). Plant spacing in each row was 35 cm with a density of 67,500 plants ha^−1^. Maize (variety Jinping 608) was sown on 26 April 2015, 21 April 2016, and 24 April 2017, and crops were harvested on 10 October, 22 September 2016, and 8 October 2017.

### Measurements

Three plants were randomly selected from each plot to collect above-ground dry matter at anthesis and maturity stages. Samples were initially oven-dried at 105°C for 30 min and then dried to constant at 70°C.


(1) $$\text{DM partitioning}(\%)=\frac{\text{Portion of DM}}{\text{Total biomass}}\times 100\%$$

Portion of DM, leaf, stem and sheath, and spike at anthesis; leaf, stem and sheath, bracts and cobs, and grain at maturity.


(2) Post−anthesis DM accumulation=DM atmaturity−DM atanthesis

(3) Translocation of DM accumulated before anthesis=DM atanthesis−accumulated non−grain DM atmaturity

(4) $$\text{DM translocation efficiency}\left(\%\right)=\frac{\text{Translocation of DM accumulated before anthesis}}{\text{Total DM at maturity}}\times 100\%$$

(5) $$\text{Contribution of pre-anthesis assimilates to grain}\left(\%\right)=\frac{\text{Translocation of DM accumulated before anthesis}}{\text{ grain dry weight}}\times 100\%$$

Plant laboratory tests were conducted after the maize harvest, including thousand kernel weight (TKW), row number per spike (RN), grain number per row (GN), and harvest index (HI). The maize grain yield was measured according to the plot at the maturity stage. Yield components (TKW, RN, and GN) were determined by randomly selecting 15 plants from each plot. The whole maize plant was taken from each plot at the flare opening, tasseling, spinning, and physiological maturity stages, the fully developed leaves (the top third leaf was taken at the flare opening stage, the top fifth leaf was taken at the tasseling stage, and the ear leaf was taken after the spinning stage) were separated, and fresh samples were frozen with liquid nitrogen and stored in a refrigerator at −80°C. The enzymatic activity of the leaves (NR, nitrate reductase; NiR, nitrite reductase; GS, glutamine synthetase; GOGAT, glutamine oxoglutarate aminotransferase) was measured using an ELISA reagent box meter (Suzhou Comin Biotechnology Co. Ltd.).


(6) $$\text{Harvest index }(\text{HI})=\frac{\text{GY}}{\text{Total above ground biomass at maturity}}$$

The soluble sugars and starch were determined according to the method described by Li et al. (2017b). Concentrations of soluble sugars and starch were quantified using a colorimetric approach with anthrone reagent at 620 nm. The non-structural carbohydrates (NSC) concentration of a specific plant part refers to the sum of the concentrations of soluble sugars and starch (mg glucose g^−1^ dry weight). Leaf samples collected at the spinning and physiological maturity stages were used to measure NSC. The various parameters related to non-structural carbohydrate movement within maize leaves were calculated according to Wu et al. (2019), as follows:


(7) $$\text{NSC translocation}(\text{kg }{\text{m}}^{-2})=\text{NSC content at anthesis}-\text{NSC content at maturity}\;$$

(8) $$\text{NSC translocation efficiency}\left(\%\right)=\frac{\text{NSC translocation}}{\text{NSC content at anthesis}}$$

Plant N content was determined by the Kjeldahl method using each portion of DM at the anthesis and maturity stages, and the grain protein concentration (GPC) was calculated by multiplying the total grain N concentration by 5.7 (Chu et al., 2023). The following equation was used to estimate the parameters of nitrogen translocation, distribution, and NUE in maize plants (Spyridon et al., 2012; Fotiadis et al., 2017; Dhillon et al., 2018):


(9) N translocation=N content at anthesis−N content at maturity (excluding grain N content)

(10) $$\text{ N translocation efficiency }\left(\text{NtE}\right)=\frac{\text{N translocation}}{\text{N content at anthesis}}$$

(11) Post-anthesis N uptake (PANU)=N content at maturity−N content at anthesis

(12) $$\text{N utilization efficiency }\left(\text{NUtE}\right)=\frac{\text{Grain yield}}{\text{N uptake by the DM at maturity}}$$

(13) $$\text{N harvest index }\left(\text{NHI}\right)=\frac{\text{N in Grain yield}}{\text{N in }total\;plant\;biomass}$$

### Statistical analysis

The normality and homogeneity of variance were checked using the Shapiro–Wilk test (*P >*0.05) and Levene’s test (*P >*0.05) before statistical analysis. One-way analysis of variance (ANOVA) together with Least-Significant Difference (*P<*0.05) was performed using SPSS software (version 20.0; SPSS Inc., Chicago, IL, United States) to identify the effect of nitrate–ammonium ratio on yield components, DM accumulation, and N utilization. Fixed effects of year, nitrate–ammonium ratio treatment, and their interactions and random effects were replication and replication × year interactions. The means were compared using Duncan’s multiple range test at a probability level of 0.05. Figures were generated using Origin 2019b (Systat Software Inc.).

## Results

### Variance analysis of grain yield, DM accumulation and N use related traits

Comprehensive analysis of variance showed that all traits (grain yield, DM accumulation, and N use) were significantly affected by nitrate–ammonium ratio (except spike number, HI, and NHI) and year (except row number) (**Table 3**). There was a significant interaction between nitrate–ammonium ratio treatment and year for almost all traits (except TKW, HI, N translocation efficiency, and NHI) (**Table 3**).

**Table 3:** Variance analysis for traits related to yield components, DM accumulation and N use.

		Nitrate–ammonium ratio treatment	Year	N × Y
	df	3	2	6
Yield components	GY (grain yield) (kg ha^−1^)	***	***	**
	Spike number (per m^2^)	ns	***	**
	Row number (per spike)	***	ns	***
	Grain number (per row)	**	***	*
	Thousand kernel weight (g)	**	***	ns
DM accumulation	Total DM at anthesis (kg ha^−a^)	***	***	***
	Total DM at maturity (kg ha^−a^)	***	***	***
	Translocation of DM accumulated before anthesis (kg ha^−a^)	***	***	***
	DM translocation efficiency (%)	***	***	***
	Post-anthesis DM accumulation (kg ha^−a^)	**	***	**
	Contribution of pre-anthesis assimilates to the grain (%)	***	***	***
	HI	ns	ns	ns
N utilization	Total N content at anthesis (kg ha^−a^)	***	***	**
	Total N content at maturity (kg ha^−a^)	***	***	**
	N translocation (kg ha^−a^)	**	***	**
	N translocation efficiency (%)	*	*	ns
	Post-anthesis N uptake (kg ha^−a^)	**	**	*
	N utilization efficiency (kg kg^−g^)	***	***	**
	N harvest index (kg kg^−g^)	ns	ns	ns
	Grain protein concentration (%)	**	**	*

ns denotes P >0.05; *, **, and *** represent significance at the 0.05, 0.01 and 0.001 probability levels, respectively.

### Maize grain yield and yield components

The nitrate–ammonium ratio had a significant impact on grain yield (GY) (**Table 4**). N4 treatment had the highest GY which was 12,992.42, 6,779.28, and 5,990.22 kg ha^−1^ in 2015, 2016, and 2017, respectively, which were increased by 3.31%–9.94%, 68.6%–26.30%, and 8.292%–36.08% compared with N1, N2, and N3 treatments. In terms of yield components, the row number (RN) per spike, GN, and TKW significantly increased under the N4 treatment (**Table 4**). TRN per spike, GN per row, and TKW were positively correlated with GY (**Table 5**).

**Table 4:** The comparison in GY and yield components among different nitrate–ammonium ratio treatments (mean 3 years).

Year	Treatments	GY (kg ha^−1^)	SN (per m^2^)	RN (per spike)	GN (per row)	TKW (g)
2015	N1	11,818.18 c	8.87 a	17.67 b	37.40 a	301.30 c
	N2	12,575.76 b	8.89 a	16.67 c	35.98 a	328.40 b
	N3	12,272.73 b	8.73 a	18.00 ab	35.13 a	312.73 bc
	N4	12,992.42 a	8.94 a	20.00 a	37.40 a	348.83 a
2016	N1	5,367.67 b	4.51 b	16.00 b	27.11 b	191.27 c
	N2	6,343.84 a	4.85 ab	16.89 ab	28.44 b	204.00 b
	N3	5,751.92 b	4.70 ab	16.00 b	30.89 a	217.90 b
	N4	6,779.28 a	5.01 a	17.00 a	31.22 a	244.35 a
2017	N1	4,401.93 b	4.22 a	13.38 d	26.00 b	369.43 b
	N2	5,531.85 a	4.62 a	18.17 b	29.33 ab	362.80 b
	N3	4,789.29 b	4.51 a	17.13 c	27.63 b	363.20 b
	N4	5,990.22 a	4.84 a	21.33 a	32.67 a	383.13 a

SN, spike number; RN, row number, GN, grain number, TKW, 1,000-kernel weight. Different lowercase letters after the values in the same column indicate that the difference was significant at P<0.05 under LSD test.

**Table 5:** Correlation coefficients between DM and N accumulation, partitioning, translocation, and yield and N use efficiency of maize.

Items	GY	RN	GN	TKW	TDM	DMa	DMm	DMtE	HI	TNAm	NtE	PANU	NPFP	GPC	NHI
GY	1														
RN	.908**	1													
GN	.768**	0.566	1												
TKW	.800**	0.695*	0.821**	1											
TDM	0.410	0.340	0.425	0.587*	1										
DMa	0.420	0.388	0.300	0.192	−0.545	1									
DMm	0.942**	0.839**	0.826**	0.757**	0.299	0.573	1								
DMtE	0.170	0.179	0.127	0.379	0.816**	−0.750**	−0.066	1							
HI	0.030	−0.171	0.322	0.376	−0.056	0.111	0.069	−0.026	1						
TNAm	0.832**	0.736**	0.784**	0.314	0.558	0.889	−0.098	−0.106	0.098	1					
NtE	−0.588*	−0.585*	−0.523	−0.449	−0.049	−0.564	−0.732**	0.324	0.057	-0.690	1				
PANU	0.330	0.369	0.460	0.315	0.033	0.316	0.507	−0.229	−0.025	0.377	−.775**	1			
NPFP	1.000**	0.908**	0.768**	0.800**	0.41	0.42	0.942**	0.17	0.03	0.832*	−0.588*	0.33	1		
GPC	0.788**	0.779**	0.736**	0.782**	0.469	0.313	0.800**	0.175	−0.134	0.828**	−.750**	0.636*	0.788*	1	
NHI	−0.340	−0.252	−0.099	0.153	0.502	−0.682*	−0.392	0.610*	0.057	−0.300	0.392	−0.055	−0.343	−0.034	1

GY, grain yield (kg ha^−1^); RN, row number (per spike); GN, grain number; TKW, thousand kernel weight; TDM, translocation of DM accumulated before anthesis; DMa, post-anthesis DM accumulation; DMm, DM accumulation at maturity; DMtE, DM translocation efficiency; HI, harvest index; TNAm, total N accumulation at maturity; NtE, N translocation efficiency; PANU, post-anthesis N uptake; NUtE, N utilization efficiency; NPFP, N partial factor productivity; GPC, grain protein concentration.

*: significant at the 0.05 probability level; **: significant at the 0.01 probability level.

### Dry matter accumulation, partitioning, and translocation

The nitrate–ammonium ratio had a significant effect on DM accumulation and partitioning (**Table 6**). Mixed NO_3_^−^-N and NH_4_^+^-N increased the total DM at the anthesis and maturity stages, compared with that of NO_3_^−^-N. Different nitrate–ammonium ratios did not have the same effect on the proportion of DM in different organs at the anthesis and maturity stages; N3 treatment decreased the DM partitioning of spikes and grains at the anthesis and maturity stages in 2016 and 2017, while increasing the proportion of DM in grains at maturity in 2015, compared to N4. Compared with the N3 treatment, the N4 treatment decreased the DM partitioning of stems, promoted the DM transfer from stem to grain, and further increased the DM partitioning of spikes and grains at anthesis and maturity, which is the basis for yield formation.

**Table 6:** Effects of different years and nitrate–ammonium ratio treatment on the mean values of DM accumulation and partitioning in different organs (**Equation 1**).

Year	Treatments	The total DM and the portion of DM in different organs at anthesis (%)				The total DM and the portion of DM in different organs at maturity (%)				
		Total (kg ha^−1^)	Leaves	Stem	Spike	Total (kg ha^−1^)	Leaves	Stem	Bract	Grain
2015	N1	11,261.84 b	16.29 a	56.63 b	27.08 a	21,960.73 c	7.25 b	28.04 a	10.91 a	53.80 a
	N2	13,246.95 b	21.74 a	64.92 a	13.33 c	25,336.28 a	10.24 a	29.98 a	14.10 a	49.66 a
	N3	12,647.30 b	21.70 a	68.83 a	9.47 c	23,649.48 b	11.03 a	23.17 b	13.89 a	51.91 a
	N4	17,464.67 a	18.78 a	63.94 a	17.28 b	26,765.13 a	8.31 b	31.59 a	11.53 a	48.57 a
2016	N1	7,396.32 c	26.95 b	52.77 b	20.28 a	11,410.01 c	13.34 b	25.14 ab	14.48 a	47.04 a
	N2	7,665.89 b	32.66 a	50.39 b	16.95 b	12,345.49 b	18.45 a	20.81 b	9.36 b	51.38 a
	N3	7,538.06 b	23.78 b	59.87 a	16.35 b	13,084.65 a	16.96 ab	30.08 a	9.00 b	43.96 b
	N4	8,163.58 a	23.76 b	53.61 b	22.62 a	13,756.39 a	20.52 a	16.57 b	13.65 a	49.26 a
2017	N1	8,595.50 b	23.78 b	59.87 a	16.35 c	11,077.85 b	15.14 b	28.66 ab	16.45 a	39.76 a
	N2	9,711.60 ab	36.08 a	35.63 b	26.62 a	14,761.87 a	23.90 a	26.39 b	12.16 b	37.55 a
	N3	9,257.20 c	24.86 b	54.72 a	20.42 b	12,985.76 b	19.11 ab	32.10 a	11.86 b	36.92 a
	N4	10,262.00 a	26.95 b	52.77 a	21.95 b	14,434.45 a	23.65 a	19.09 c	15.73 a	41.53 a

Different lowercase letters after data indicate significant differences under mean values between nitrate–ammonium ratio treatments and years at P<0.05, respectively.

The nitrate–ammonium ratio also had a significant effect on the traits associated with DM (**Table 7**). The N4 treatment increased TDM, DMtE, and CPAG in 2015 and 2017, by 101.53%–659.00% and 17.04%–91.26%, increased DMtE by 78.15%–619.27% and 8.78%–42.05%, and increased CPAG by 90.29%–633.29% and 11.93%–21.654%, respectively, compared to N1, N2, and N3, respectively. The N4 treatment decreased DMa in 2015 compared to the other treatments. There was an insignificant difference in HI among the different nitrate–ammonium ratio treatments. Total DM accumulation (maturity) was positively correlated with GY, RN, GN, and TKW under the N treatments. In addition, a positive correlation was observed between translocation of DM and TKW (**Table 5**).

**Table 7:** Mean values of DM translocation parameters was influenced by the growing season and nitrate–ammonium ratio treatment (**Equations 2**–**6**).

Year	Treatments	TDM (kg ha^−1^)	DMtE (%)	DMa (kg ha^−1^)	CPAG (%)	HI
2015	N1	1,671.10 b	7.58 b	10,698.89 b	14.04 b	0.54
	N2	486.43 c	1.92 c	12,089.33 a	3.87 c	0.50
	N3	1,832.00 b	7.75 b	11,002.18 ab	14.93 b	0.52
	N4	3,691.97 a	13.81 a	9,300.46 c	28.41 a	0.49
2016	N1	1,353.97 a	10.88 a	4,013.70 c	20.26 a	0.47
	N2	1,373.45 a	9.11 b	4,679.60 b	21.64 a	0.51
	N3	910.12 c	11.14 a	5,546.59 a	16.12 b	0.44
	N4	1,045.44 b	8.20 b	5,592.80 a	18.68 ab	0.49
2017	N1	1,919.58 b	14.31 a	2,482.35 c	33.51 b	0.40
	N2	1,174.64 c	10.96 b	5,050.27 a	31.22 bc	0.38
	N3	1,467.20 c	11.37 b	3,728.56 b	30.83 c	0.37
	N4	2,246.67 a	15.57 a	4,172.45 b	37.51 a	0.42

TDM, translocation of DM before anthesis; DMtE, DM translocation efficiency; DMa, post-anthesis DM accumulation; CPAG, contribution of pre-anthesis assimilates to the grain; HI, harvest index. Different lowercase letters in the table indicate significant differences under mean values between nitrate–ammonium ratio treatments and years at P<0.05, respectively.

### The enzymatic activity of nitrogen metabolism at five developmental stages

**Figure 3:**
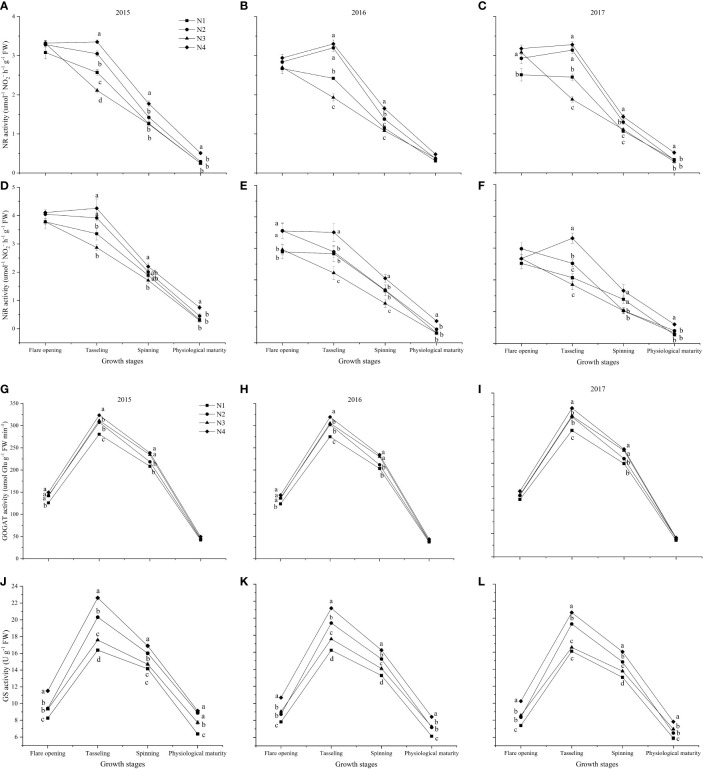
The enzymatic activity of nitrogen metabolism among different nitrate–ammonium ratio treatments. Different lowercase letters in the same column indicate significant differences among different treatments at *P*<0.05. **(A–C)**: NR activity at five developmental stages in 2015, 2016, and 2017, respectively. **(D–F)**: NiR activity at five developmental stages in 2015, 2016, and 2017, respectively. **(G–I)**: GOGAT activity at five developmental stages in 2015, 2016, and 2017, respectively. **(J–L)**: GS activity at five developmental stages in 2015, 2016, and 2017, respectively.

The enzymatic activity of NR and NiR decreased under the N1 and N3 treatments, but GOGAT and GS increased first and then decreased under the different nitrate–ammonium ratio treatments from the flare opening to physiological maturity stages (**Figure 3**). N4 treatment increased the activity of NR, NiR, and GS at tasseling, spinning, and physiological maturity, respectively (**Figures 3A–F, J, L**). Compared with the single application of nitrate nitrogen, the mixed supply of nitrate and ammonium nitrogen significantly increased GOGAT activity at the flare opening and tasseling stages (**Figures 3G–I**). Compared with other nitrate–ammonium ratio treatments, N4 increased the average enzyme activity during the maize growth period, NR, NiR, GOGAT, and GS increased by 9.30%–32.82%, 13.19%–37.94%, 4.11%–16.00%, and 11.19%–30.82%, respectively.

### Changes in non-structural carbohydrates after anthesis

**Figure 4:**
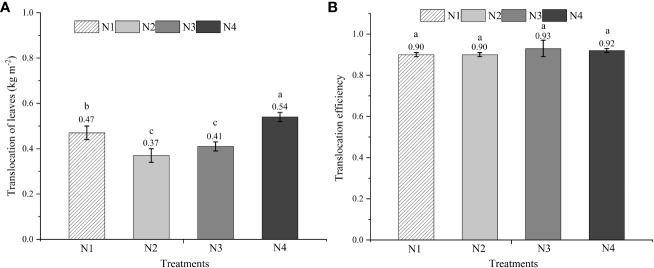
The amount and efficiency of total non-structural carbohydrate (TNSC) translocation of leaves. Different lowercase letters above the vertical bar indicate significant differences among different treatments at *P<*0.05. (**Equations 7**, **8**). **(A)** The translocation amount of total non-structural carbonhydrate (TNSC) of leaves. **(B)**: The translocation efficiency of total non-structural carbonhydrate (TNSC) of leaves.

The mean non-structural carbohydrates accumulation of functional leaves at anthesis was about 0.54 kg per m^2^ in the N4 treatment, compared with 0.47 kg per m^2^ in the N1 treatment, 0.37 kg per m^2^ in the N2 treatment, and 0.41 kg per m^2^ in the N3 treatment (**Figure 4A**). No significant difference was observed in the non-structural carbohydrate translocation efficiency among the four N formula treatments (**Figure 4B**).

### N accumulation and partitioning

The nitrate–ammonium ratio treatment had a significant effect on the total N accumulation and partitioning of different organs (**Table 8**). The N1 treatment significantly reduced the total nitrogen accumulation; the anthesis decreased by 32.52%, 16.24%, and 49.48%, and the maturity stage decreased by 26.70%, 39.19%, and 35.80%, respectively, compared to the N1 treatments. Compared with that of the N1 treatment, the N portion distributed to the leaves increased under N4 treatments at both stages, whereas that distributed to the stem decreased at anthesis. N4 treatment increased nitrogen distribution to spike at anthesis and to grain at maturity stage compared to the N2 treatment, and N2 treatment had the lowest nitrogen distribution to spike and grain at anthesis and maturity stages.

**Table 8:** Mean values of total N accumulation and distribution in different organs at anthesis and maturity stages was influenced by the growing season and nitrate–ammonium ratio treatments.

Year	Treat ments	Total N and the portion of N in different organs at anthesis (%)				Total N and the portion of N in different organs at maturity (%)				
		Total (kg ha^−1^)	Leaves	Stem	Spike	Total (kg ha^−1^)	Leaves	Stem	Bract	Grain
2015	N1	101.30 c	32.94 b	41.69 b	25.37 a	177.18 b	7.65 b	14.09 b	7.70 b	70.55 a
	N2	150.13 a	48.55 a	40.06 b	11.40 c	261.02 a	7.88 b	24.36 a	8.99 b	58.77 c
	N3	141.39 b	42.74 a	48.82 a	8.44 c	243.02 a	9.06 a	7.46 c	7.65 b	75.83 a
	N4	154.41 a	47.29 a	33.27 b	19.45 b	241.71 a	8.78 a	12.91 b	11.87 a	66.45 b
2016	N1	103.42 b	44.40 a	35.32 ab	20.28 b	146.81 c	10.08 b	9.10 b	14.38 a	66.43 a
	N2	123.47 a	45.55 a	36.09 ab	18.36 b	186.76 b	13.88 a	8.24 b	9.48 b	68.40 a
	N3	96.38 b	35.07 b	40.70 a	24.23 a	209.96 b	13.30 a	16.55 a	7.69 b	62.45 a
	N4	129.16 a	44.73 a	30.06 b	25.20 a	241.42 a	14.76 a	4.68 c	10.79 b	69.77 a
2017	N1	98.31 d	30.76 c	54.95	14.29 b	138.91 c	11.22 c	10.70 b	13.32 a	64.75 a
	N2	162.57 b	53.35 a	26.13	20.52 a	177.19 b	20.59 a	16.35 a	12.29 a	50.76 b
	N3	132.48 c	29.71 c	48.08	22.21 a	158.00 b	15.36 b	14.75 a	11.51 a	58.37 ab
	N4	194.59 a	39.13 b	40.07	20.80 a	216.35 a	18.00 a	8.00 b	13.34 a	60.66 a

Different lowercase letters after data indicate significant differences under mean values between nitrate–ammonium ratio treatments and years at P<0.05, respectively.

### N utilization efficiency-related traits

The nitrate–ammonium ratio treatment had a significant effect on Nt, NtE, NPFP, and GPC, and there were no significant differences in NPFP and NHI (**Table 9**). The effects of nitrate–ammonium ratio treatment on N utilization traits were not consistent across different years. There was a significant difference between N1 and N4 in Nt and PANU in 2015 and 2017 but not in 2016. Compared with the single application of nitrate nitrogen, the mixed supply of nitrate and ammonium nitrogen resulted in lower NUtE and higher grain protein concentrations in 2015.

**Table 9:** Values of N uptake and N utilization efficiency-related traits were influenced by the growing season and nitrate–ammonium ratio treatments (**Equations 9**–**13**).

Year	Treatments	Nt (kg ha^−1^)	NtE (%)	PANU (kg ha^−1^)	NUtE (kg kg^−1^)	NPFP (kg kg^−1^)	NHI (kg kg^−1^)	GPC (%)
2015	N1	49.81 b	48.35 b	75.87 c	67.50 a	48.35	0.71	7.16 c
	N2	42.30 b	26.08 c	110.88 a	48.30 b	43.99	0.59	8.73 b
	N3	82.66 a	58.47 a	101.63 ab	50.61 b	47.42	0.76	10.50 a
	N4	72.68 a	47.44 b	87.30 b	54.06 b	46.19	0.66	9.12 b
2016	N1	53.93 b	52.34 a	43.39 c	36.65	17.89	0.66	5.55 c
	N2	64.60 a	52.12 a	63.30 b	34.16	21.15	0.68	7.29 b
	N3	17.71 c	18.35 c	113.58 a	27.43	19.17	0.62	7.48 b
	N4	56.20 b	43.34 b	112.26 a	28.08	22.60	0.70	9.60 a
2017	N1	49.44 d	50.24 ab	40.59 a	31.76	14.67	0.65	5.13 b
	N2	75.43 b	45.31 b	14.62 c	31.27	18.44	0.51	5.13 b
	N3	66.72 c	50.28 ab	25.52 b	30.48	15.96	0.58	5.26 b
	N4	109.38 a	56.30 a	21.76 bc	27.71	19.97	0.61	7.47 a

Nt, nitrogen translocation; NtE, nitrogen translocation efficiency; PANU, post-anthesis N uptake; NUtE, nitrogen utilization efficiency; NPFP, nitrogen partial fertilizer productivity; NHI, nitrogen harvest index; GPC, grain protein concentration. Different lowercase letters in the table indicate significant differences under mean values among nitrate–ammonium ratio treatments and years at P<0.05, respectively.

**Figure 5:**
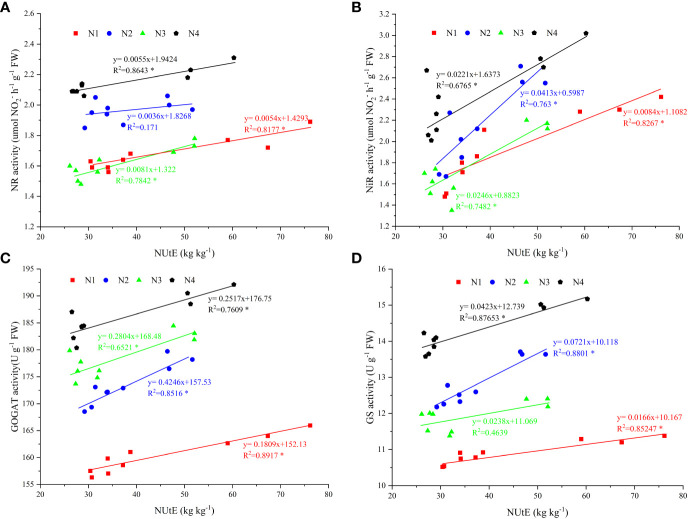
Enzymatic activity of nitrogen metabolism and its relationship with N use efficiency under four nitrate–ammonium ratio treatments. * and ** represent significance at the 0.05 and 0.01 probability levels, respectively. The data points are the average values of enzymatic activity of N metabolism and N use efficiency during three growing seasons. The X-axis shows NUtE and the Y-axis shows the enzymatic activity of the N metabolism value. **(A–D)**: Relationship between NR activity, NiR activity, GOGAT activity, GS activity, and nitrogen fertilizer use efficiency, respectively.

**Figure 6:**
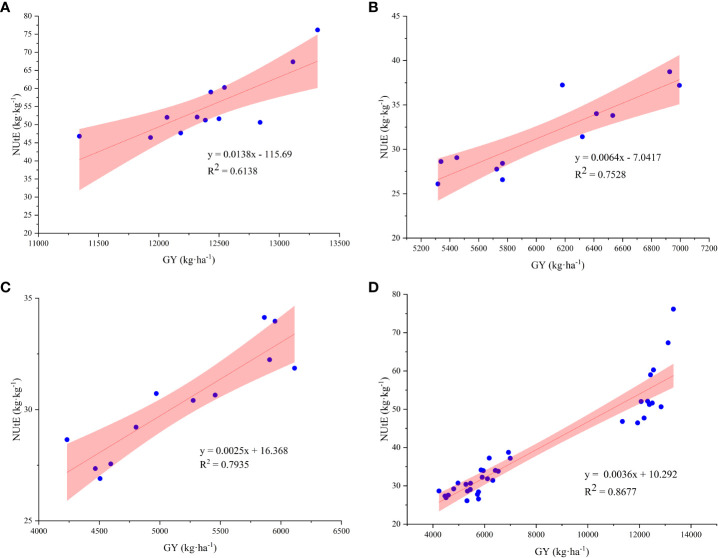
Correlation relationship between GY and NUtE among the different nitrate–ammonium ratio treatments. * and ** represent significance at the 0.05 and 0.01 probability levels, respectively. The X-axis shows GY, and the Y-axis shows the NUtE value. **(A–C)**: Correlation relationship between GY and NUtE in 2015, 2016, and 2017, respectively. **(D)** Correlation relationship between GY and NUtE in average three growing seasons.

The NUtE and activities of NR, NiR, GOGAT, and GS under N4 treatment were significantly positively correlated, with R^2^ values of 0.8643, 0.6765, 0.7609, and 0.8765, respectively (**Figures 5A–D**). In addition, the results of the three-year study showed that both GY and NUtE were significantly and positively correlated (**Figure 6**). Among these N-related parameters, under nitrate–ammonium ratio treatment, NtE was negatively correlated with GY, RN, and PANU; GPC was negatively correlated with NtE. In contrast, NPFP was positively correlated with GY, RN, GN, TKW, and total N accumulation at maturity (TNAm) under nitrate–ammonium ratio treatments, and GPC was positively correlated with GY, RN, GN, TKW, and PANU under nitrate–ammonium ratio treatments (**Table 5**).

## Discussion

### Effect of nitrate–ammonium ratio on grain yield and yield-related traits

A combination of NO_3_^−^-N and NH_4_^+^-N showed a significantly higher grain yield potential than NO_3_^−^-N or NH_4_^+^-N in maize (George et al., 2016; Li et al., 2017a, 2018; Wang et al., 2018; Zhang et al., 2019). However, previous studies on the effects of NO_3_^−^-N and NH_4_^+^-N on maize have yielded different results. George et al. (2016) reported that except for NO_3_^−^, agriculturally relevant proportions of NH_4_^+^ supplied can increase growth and grain yield of maize. Wang et al. (2018) reported that in the well-aerate dryland soil, NO_3_^−^-N was the main form of N uptake by maize, and application of NO_3_^−^-N relative to NH_4_^+^-N increased maize DM. In the present study, NO_3_^-^/NH_4_^+^ with 3:1 ration significantly increased the grain yield compared with that of single NO_3_^−^-N supply treatment (**Table 4**), probably because the appropriate addition of NH_4_^+^ to NO_3_^−^ supply can promote the absorption of N to promote the growth of maize (George et al., 2016). In maize production, the main determinant of yield is the total kernel number at harvest (Ma et al., 2015), and the most recommended management methods for maize have been focused on maximizing the number of grains per unit area (Bonelli et al., 2016). Our results highlight that the yield advantage of N4 over the other nitrate–ammonium ratio treatments was due to the higher row per spike and GN per row.

Different NO_3_^−^/NH_4_^+^ ratios in N supply can influence the rate of plant growth and DM allocation (Chang et al., 2010). DM production is directly related to the nitrate–ammonium ratio, and photosynthetic performance was improved by increasing the proportion of NO_3_^−^-N, which then promoted yield formation (Li et al., 2017a). In the present study, maize demonstrated a considerable advantage in DM accumulation under N4 treatment. Furthermore, a significant correlation between DM accumulation at maturity and GY, RN, GN, and TKW was observed (**Table 5**), which agrees with the results of previous studies (Liu et al., 2014; Li et al., 2017a; Cai et al., 2022).

DM allocation is the product of the flow of assimilates from the source organs (leaves and stems) along the transport route to the storage organs (grains) (Amanullah et al., 2021), which is closely related to the availability at anthesis (Liu et al., 2020). In the present study, N4 treatment reduced the production of maize assimilate at the grain-filling stage, which directly decreased DM accumulation post-anthesis (**Table 6**). It is noteworthy that the CPAG was significantly increased under the N4 treatment, which could be attributed to the low rate of photosynthesis, which leads to a low supply of post-anthesis assimilates for grain filling, thereby increasing the demand for the translocation of dry matter accumulated before flowering (Ercoli et al., 2008). Overall, NO_3_^−^/NH_4_^+^ ratios with 3:1 treatment showed advantages in total DM accumulation at both the anthesis and maturity stages, translocation of DM accumulation before anthesis, and CPAG.

### Effect of nitrate–ammonium ratio on enzymatic activity and non-structural carbohydrate translocation

The current study showed that N4 treatment had higher enzymatic activity (NR, NiR, GOGAT, and GS) at the flare opening to the physiological maturity stage (**Figure 3**), indicating that NO_3_^-^/NH_4_^+^ ratios of 3:1 improved post-anthesis assimilate accumulation by maintaining higher activity of nitrogen-metabolizing enzymes, and finally resulted in increased DM translocation. Prey et al. (2019) reported that GY was more connected with post-anthesis assimilation, also DM translocation contributed appreciably to GY by 31%–44%. Therefore, it is necessary to maintain assimilate accumulation post-anthesis by improving leaf enzymatic activity and delaying leaf senescence (Liu et al., 2020). These findings imply that the NO_3_^−^/NH_4_^+^ ratios with 3:1 treatment showed a higher enzymatic activity than NO_3_^−^/NH_4_^+^ ratios with 1:0, 1:1, and 1:3 treatments, which improved the GY of maize.

Suitable N (forms, amount, or application time) boosted the NSC reserves accumulated in stem pre-anthesis (Liang et al., 2017b). NSC allocation from leaves to harvested sinks is a major predictor of grain yield in maize, particularly after anthesis (Liang et al., 2019). The current findings showed that N4 treatment had the highest NSC translocation in the stem, while nitrate–ammonium ratio had insignificant effects on NSC translocation efficiency. There is a highly positive correlation between GY and NSC transportation efficiency from the flowering to maturity stage under nitrogen stress (Liu et al., 2020). However, our findings showed that GY was not associated with NSC translocation efficiency, which may be related to the N supply and formula, all of which had the same total N application, except for different ratios of NO_3_^−^-N and NH_4_^+^-N. It has also been reported that the translocation of NSC accumulated in the stem post-anthesis to the grains increased under N stress compared with sufficient N supply (Liu et al., 2020).

### High-yielding trait of NO_3_^−^/NH_4_^+^ with 3:1 may be associated with high NtE and NPFP

The mixed NO_3_^−^-N and NH_4_^+^-N application improved the total N content in the leaf, stem, sheath, and spike at anthesis, promoted the N transportation of source organs during the grain-filling stage, and decreased the total N content of source organs at the maturity stage compared to the sole NO_3_^−^-N or NH_4_^+^-N (Huang et al., 2013). In the current study, NO_3_^−^/NH_4_^+^ ratios with 3:1 treatment decreased N partitioning to the stem + sheath, but increased N partitioning to the spike at anthesis (**Table 8**). It is generally believed that N accumulated before anthesis is the major source of grain N (Gaju et al., 2014), and our results also support this conclusion. The N distribution of vegetative portions (stem + sheath and leaves) at the maturity stage was considerably lower than that at anthesis, which indicated that N4 treatment increased nitrogen redistribution at the maturity stage of maize. The mixed application of NO_3_^−^-N and NH_4_^+^-N improved the nitrogen uptake and utilization of maize and achieved the highest nitrogen accumulation during the entire maize growth period (Li et al., 2018). The present results demonstrated that the mixed application of NO_3_^−^-N and NH_4_^+^-N significantly increased N accumulation compared to the single NO_3_^−^-N treatment.

The uptake and translocation of N by plants are generally balanced, and the most effective way to increase nitrogen use efficiency is to increase plant N uptake and N translocation (Wang et al., 2020). Previous studies have shown that nitrogen translocation is positively correlated with maize GY (Liu et al., 2015, 2019). NO_3_^−^/NH_4_^+^ with 3:1 significantly boosted N translocation in the stems and leaves of maize, and approximately 50% and 60% of the nitrogen in stems and leaves were transferred to the grain, respectively, compared with sole NO_3_^−^-N supply (Li et al., 2018). In the current study, N translocation increased with increasing NO_3_^−^-N ratios (**Table 9**). Accordingly, NO_3_^−^/NH_4_^+^ ratio of 3:1 significantly improved the N translocation efficiency and ensured the normal supply of maize grain nitrogen demand, which is consistent with previously published studies (Wang et al., 2012).

Increasing the N utilization rate is a common concern among agricultural workers, and there are many factors that affect N utilization; therefore, N utilization is the result of the interaction of many factors. A study on wheat (Zhengmai 366) showed that NO_3_^−^/NH_4_^+^ at 75:25 provided an approximately 10%–22% increase in nitrogen use efficiency above that of other NO_3_^−^/NH_4_^+^ ratios (Huang et al., 2013). Daba et al. (2021) indicated that both NPFP and agronomic efficiency are negatively correlated with an increase in N application. In the present study, the mixed supply of nitrate and ammonium nitrogen resulted in significantly higher N translocation, post-anthesis N uptake, and N partial factor productivity, all of which set the stage for increased maize yields.

## Conclusions

NO_3_^−^/NH_4_^+^ with 3:1 showed advantages in grain yield and most grain yield-related traits, enzymatic activity of nitrogen metabolism (NR, NiR, GS, and GOGAT), and non-structural carbohydrate accumulation compared to NO_3_^−^/NH_4_^+^ with 1:0, 1:1, and 1:3 treatments. The results paved the way to lay the foundation for improving the N utilization efficiency. NO_3_^−^/NH_4_^+^ at a ratio of 3:1 strongly promoted the accumulation of dry matter and N in vegetative organs transferred to reproductive organs and improved the pre-anthesis dry matter and nitrogen translocation efficiency. Overall, a NO_3_^−^/NH_4_^+^ ratio of 3:1 is recommended for high-yield and sustainable maize management strategies in Northwestern China.

## Data availability statement

The raw data supporting the conclusions of this article will be made available by the authors, without undue reservation.

## Author contributions

BW: Writing – original draft. ZC: Investigation, Data curation, Software, Writing – review & editing. EZ: Writing – review & editing. LG: Conceptualization, Methodology, Writing – review & editing. YG: Conceptualization, Funding acquisition, Methodology, Writing – review & editing. BY: Writing – review & editing. HL: Data curation, Resources, Writing – review & editing. YW: Data curation, Writing – review & editing. HW: Data curation, Writing – review & editing. LL: Data curation, Writing – review & editing.
