# Supplementary material for: Post-anthesis dry matter and nitrogen accumulation, partitioning, and translocation in maize under different nitrate–ammonium ratios in Northwestern China
Source: Front Plant Sci. 2024 Mar 19;15:1257882. doi: 10.3389/fpls.2024.1257882 (PMC10985316; doi:10.3389/fpls.2024.1257882)
Supplement: Supplementary file 1 [file Table_1.docx]

**TABLE S1 |** **Details of the treatments in 2015**

| Treatment | Symbol | Fertilizer amount | | | DMPP |
| --- | --- | --- | --- | --- | --- |
|  |  | KNO_3_ | (NH_4_)_2_SO_4_ | K_2_SO_4_ | (kg ha^-1^)^‡^ |
|  |  | (kg ha^-1^) | (kg ha^-1^) | (kg ha^-1^) |  |
| KNO_3_ fertilizer | N1 | 1332.9 | 0 | 0 | 0 |
| KNO_3_:(NH_4_)_2_SO_4_ with ratio^†^ 1:1 | N2 | 666.5 | 424.35 | 573.95 | 0.9 |
| KNO_3_:(NH_4_)_2_SO_4_ with ratio^†^ 1:3 | N3 | 333.25 | 636.5 | 860.95 | 1.35 |
| KNO_3_:(NH_4_)_2_SO_4_with ratio^†^ 3:1 | N4 | 999.68 | 212.16 | 287 | 0.45 |

† ratios are given as NO_3_^-^/NH_4_^+^ and refer to pure N content, ‡ The application of DMPP was calculated as 1% of pure N in NH_4_^+^-N content of the basic fertilizer.

**TABLE S2 |** **Fertilizer application amount at different period in 2015**

| Treatment | KNO_3_ (N 13.5%) (kg ha-1) | | | (NH_4_)_2_SO_4_ (N 21.21%) (kg ha-1) | | | K_2_SO_4_ (kg ha-1) | | | DMPP (kg ha-1) | | |
| --- | --- | --- | --- | --- | --- | --- | --- | --- | --- | --- | --- | --- |
|  | Before sowing | Jointing | Filling | Before sowing | Jointing | Filling | Before sowing | Jointing | Filling | Before sowing | Jointing | Filling |
| N1 | 799.74 | 266.58 | 266.58 | 0 | 0 | 0 | 0 | 0 | 0 | 0 | 0 | 0 |
| N2 | 399.90 | 133.30 | 133.30 | 254.61 | 84.87 | 84.87 | 344.37 | 114.79 | 114.79 | 0.54 | 0.18 | 0.18 |
| N3 | 199.95 | 66.65 | 66.65 | 381.90 | 127.30 | 127.30 | 516.57 | 172.19 | 172.19 | 0.81 | 0.27 | 0.27 |
| N4 | 599.81 | 199.94 | 199.94 | 127.30 | 42.43 | 42.43 | 172.20 | 57.40 | 57.40 | 0.27 | 0.09 | 0.09 |

DMPP, 3,4-Dimethylpyrazole phosphate.
